# Health Beliefs and Mediterranean Diet Adherence: Key Predictors of Dietary Behavior for Dementia Prevention

**DOI:** 10.1093/geroni/igaf122.2523

**Published:** 2025-12-31

**Authors:** Offer Edelstein

**Affiliations:** Ben Gurion University of the Negev, Be’er Sheva, HaDarom, Israel

## Abstract

**Background:**

Dementia prevention strategies emphasize modifiable lifestyle factors, with adherence to the Mediterranean diet (MD) being a key recommendation. This study sought to (i) assess MD adherence among Israeli-born adults aged 50 and older and (ii) explore how Health Belief Model (HBM) constructs (Rosenstock, 1966, 1974) relate to dietary adherence.

**Method:**

A cross-sectional online survey was conducted in 2022–2024 with a convenience sample of 895 Israeli-born individuals aged 50 and above. MD adherence was measured using the Israeli Mediterranean Diet Adherence Screener (I-MEDAS) (Abu-Saad et al., 2018), and cognitive perceptions were evaluated using the Motivation to Change Lifestyle and Health Behaviors for Dementia Risk Reduction questionnaire (Kim et al., 2014).

**Results:**

The mean MD adherence score was 10.11 (SD = 1.36) out of 17. Multivariate regression analysis identified perceived barriers (β = -0.301, p < .001), cues to action (β = 0.233, p < .001), and perceived severity (β = 0.191, p < .001) as significant predictors of dietary adherence. Additionally, being female (β = 0.212, p < .001) and having a lower income (β = -0.151, p < .05) were also associated with adherence. The model accounted for 22.4% of the variance in MD adherence [F(5,892) = 15.56, p < .0001].

**Conclusions:**

These findings suggest that addressing perceptions of disease severity and perceived barriers, as well as increasing cues to action, may enhance adherence to the MD. Public health interventions should incorporate these insights to promote dietary behavior change in midlife and older adults.

